# Siberian Subtype Tickborne Encephalitis Virus, Finland

**DOI:** 10.3201/eid1210.060320

**Published:** 2006-10

**Authors:** Anu E. Jääskeläinen, Tapani Tikkakoski, Nathalie Y. Uzcátegui, Andrey N. Alekseev, Antti Vaheri, Olli Vapalahti

**Affiliations:** * Haartman Institute, University of Helsinki, Helsinki, Finland;; † Keski-Pohjanmaa Central Hospital, Kokkola, Finland;; ‡ Russian Academy of Sciences, St. Petersburg, Russia;; § HUSLAB Hospital District of Helsinki and Uusimaa, Helsinki, Finland;; ¶ Faculty of Veterinary Medicine, University of Helsinki, Helsinki, Finland

**Keywords:** Tickborne encephalitis virus, Ixodes ricinus, Ixodes persulcatus, Siberian subtype TBEV, dispatch

## Abstract

We isolated 11 Siberian subtype tickborne encephalitis virus (TBEV) strains from *Ixodes persulcatus* ticks from a TBEV-endemic focus in the Kokkola Archipelago, western Finland. Thus *I. persulcatus* and the Siberian TBEV are reported in a focus considerably northwest of their previously known range in eastern Europe and Siberia.

Tickborne encephalitis (TBE) is a disease endemic in a zone extending from central and eastern Europe to Siberia and Japan. Three subtypes of the causative agent tickborne encephalitis virus (TBEV) are known: the European, Siberian, and Far Eastern (*1**,**2*). The main vector for the European subtype is *Ixodes ricinus,* and for the other 2 subtypes, *I. persulcatus* (*1**,**3**–**5*). *I. ricinus* is found in Europe and Middle East (*6*), and *I. persulcatus* ranges from eastern Europe to China and Japan. The boundary between their distribution lies at the Russian side of the Finnish-Russian border (*1**,**7*). The distribution areas of both tick species overlap in eastern Europe (*4**,**5*) (Figure 1). *I. persulcatus* has not been reported from northern or western Europe except for an engorged nymph on a willow warbler (*Phylloscopus trochilus*) in northeastern Sweden in May 1992 (*6*).

**Figure 1:**
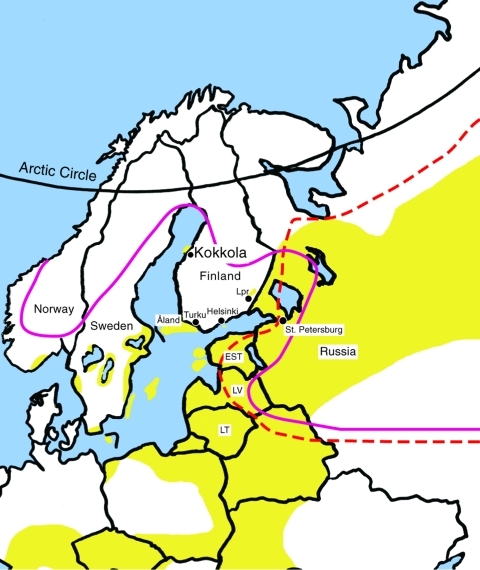
The known distribution of tickborne encephalitis (TBE)–virus endemic areas and *Ixodes* ticks in northern Europe. Yellow: TBE-endemic areas, adapted from International Scientific Working Group on Tick-Borne Encephalitis (*8*). To the south and west from the solid line, *Ixodes ricinus* distribution; to the east from the dashed line, *I. persulcatus* distribution; Lpr, Lappeenranta; EST, Estonia; LV, Latvia; LT, Lithuania.

In Finland, the TBE-endemic areas are mostly in the coastal regions: two thirds of cases come from the Åland Islands. Other TBE-endemic regions include the Archipelago of Turku, a focus in Isosaari (an island outside Helsinki), the Lappeenranta region in southeastern Finland, and the Archipelago of Kokkola in western Finland (*9*). The virus was found in the same areas (except for Isosaari) as early as the 1960s by screening antibodies to TBEV from cattle sera (*10*).

The TBE focus in the Archipelago of Kokkola (63°50´N, 23°10´E), ≈300 km south of the Arctic Circle, has a peculiar location; it is an isolated focus, far from other TBE-endemic areas, and is the northernmost TBE-endemic area known. Furthermore, the recent TBE cases have been severe with sequelae (*11**,**12*). A cluster of cases in 2002 led us to study ticks in the Kokkola Archipelago for TBEV.

## The Study

A total of 1,181 ticks were collected by flagging in the Archipelago of Kokkola in June 2004 (Table 1). In the tick-collecting areas 1–7, TBE patients had reported tick bites, and in areas 8–10, no TBE cases have been found. All the locations were islands or peninsulas within 20 km of each other.

**Table 1:** Tick collection sites in Kokkola Archipelago, June 2004*

Location	TBE case-patient, sex/age/y	No. ticks (n/f/m)	No. tick pools	Tick pools positive in RT-PCR†	Tick pools positive in virus isolation†
1	M/48/2004 (*12*)	184 (19/87/78)	19	4, 8, 9	8, 9
2	M/21/2002 (*11*)	80 (24/25/31)	8	25, 26	25, 26
3	F/24/2002 (*11*)	158 (2/87/69)	16	39	39
4	F/12/2002 (*11*)	474 (16/227/231)	48	79, 81, 84, 85, 86, 102, 118	79, 81, 84, 86, 102, 118
5	F/73/2002 (*11*)	41 (2/22/17)	5	–	ND
6	M/6/2003	166 (6/83/77)	17	–	ND
7	M/7/2003	6 (0/3/3)	1	–	ND
8	No known cases	55 (1/25/29)	6	–	ND
9	No known cases	9 (2/5/2)	1	–	ND
10	No known cases	8 (0/6/2)	1	–	ND

*TBE, tickborne encephalitis; RT-PCR, reverse transcription–polymerase chain reaction; n, nymphs; f, adult females; m, adult males; ND, not determined. †Nos. indicate the tick pool numbers, the same as used in the phylogenetic tree.

The ticks were homogenized in pools of ≈10 with Dulbecco phosphate-buffered saline plus 0.2% bovine serum albumin (D-PBS-BSA) and sand to 122 pools. RNA was isolated from 100 μL of the pools by TriPure Isolation Reagent (Roche Diagnostics, Espoo, Finland). The RNA was dissolved in 20 μL diethyl pyrocarbonate–treated water, and 10 μL was used for nested reverse transcription (RT)–PCR, amplifying a 252-nt sequence from the TBEV-NS5 gene to detect TBEV-RNA according to Puchhammer-Stöckl et al. (*13*), except that the outer forward primer used was 5´-ggaggctgaacaactgcac-3´. TBEV-RNA was detected in 13 pools (each consisted of 10 adult ticks) (Table 1). Assuming that only 1 tick in a positive pool was positive for TBEV RNA, the overall TBEV prevalence was 1.1%.

To isolate TBEV strains from the RT-PCR–positive tick pools, 20 μL of the supernatant of the pools diluted 1:1 in D-PBS-BSA was injected intracerebrally into suckling NMRI mice. One litter of suckling mice was used for each pool. The mice were followed for 14 days or until symptoms of illness appeared, and then they were killed. From 200 μL of the homogenized mouse brains, diluted 1:5 with D-PBS-BSA, RNA was extracted by TriPure, and RT-PCR for the partial TBEV-E gene was performed. The cDNA was produced with the reverse primer 5´-ccyccagccargagraagc-3´ by M-MuLV-RT enzyme (Fermentas, Vilnius, Lithuania), and subsequent PCR was performed with this and a forward primer 5´-aacagggaytttgtcactggyactc-3´ by *Taq* DNA polymerase (Fermentas) (detailed RT-PCR protocol available from the authors upon request).

**Figure 2:**
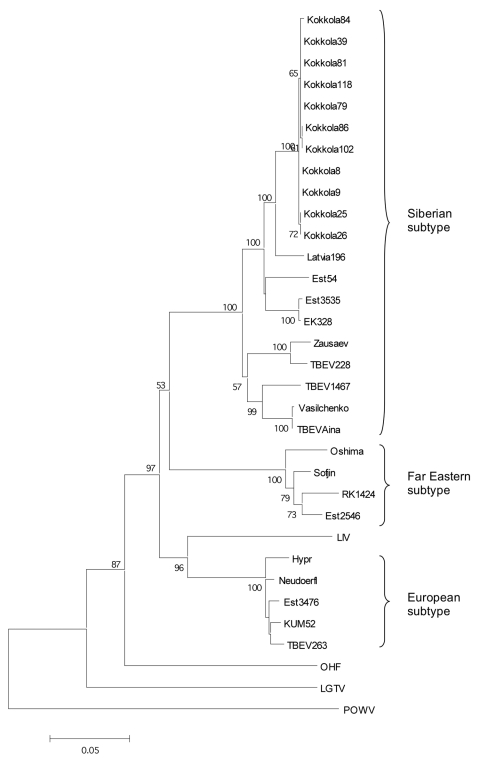
Maximum likelihood phylogenetic tree of partial E gene (1,076 nt). The bar below indicates the nucleotide substitutions per site. The accession nos. of the strains used can be seen in Table 2. The bootstrap support values <50 are not shown.

A region of 205 nt from the NS5 gene from the RT-PCR–positive tick pools and 1,225-nt stretch from the E gene from the brains of the infected suckling mice were sequenced (GenBank accession nos. in Table 2). Unexpectedly, based on the partial NS5 sequences from the RT-PCR–positive tick pools (data not shown), the TBEV strains in Kokkola belonged to the Siberian subtype of TBEV. A phylogenetic tree based on the partial E gene sequences (1,076 nt) obtained from the TBEV isolates was prepared by the maximum likelihood method (Figure 2, scripts and datasets available from the authors upon request). Within the 1,076-nt stretch of the E gene, the Kokkola strains were >99.6% identical to each other. The closest relatives were Latvia-1-96 (97% identical) and the Estonian strains Est54, Est3535, and EK328 (95%–96%). Consequently, the Siberian subtype strains isolated from Finland and nearby Baltic states form a lineage together within the Siberian subtype. Other Siberian subtype strains Vasilchenko, Aina, Zausaev, and TBEV228 showed 92%–94% identity, and the European and Far Eastern subtypes showed 84%–86% identity. However, the vector tick species for Siberian-type TBEV, *I. persulcatus,* was not known to exist in Finland. This knowledge led us to study the tick species more carefully. DNA was isolated from 20 tick pools by TriPure and resuspended in 100 μL of TE (Tris-HCl 10 mmol/L, EDTA 1 mmol/L, pH ≈8). The tick species was determined as *I. persulcatus* by amplifying an average of 339 bp from mitochondrial 16S RNA gene by PCR and subsequent sequencing according to Caporale et al. (*14*). Because the ticks were pooled and homogenized before species identification, 30 adult ticks from the same region collected later in the summer were examined microscopically. All these specimens were *I. persulcatus* by morphologic criteria.

**Table 2:** TBE virus strains compared by sequence analysis*

Strain	Geographic origin	GenBank accession no.
Kokkola 4	Location 1, Kokkola	DQ451297†
Kokkola 8	Location 1, Kokkola	DQ451298,† DQ451286‡
Kokkola 9	Location 1, Kokkola	DQ451299,† DQ451287‡
Kokkola 25	Location 2, Kokkola	DQ451300,† ^,^DQ451288‡
Kokkola 26	Location 2, Kokkola	DQ451301,† DQ451289‡
Kokkola 39	Location 3, Kokkola	DQ451302,† DQ451290‡
Kokkola 79	Location 4, Kokkola	DQ451303†DQ451291‡
Kokkola 81	Location 4, Kokkola	DQ451304,†DQ451292‡
Kokkola 84	Location 4, Kokkola	DQ451305,†DQ451293‡
Kokkola 85	Location 4, Kokkola	DQ451306†
Kokkola 86	Location 4, Kokkola	DQ451307,†DQ451294‡
Kokkola 102	Location 4, Kokkola	DQ451308,†DQ451295‡
Kokkola 118	Location 4, Kokkola	DQ451309,†DQ451296‡
Iso40	Isosaari, Finland	AJ298323
Kumlinge A 52	Åland, Finland	X60286
Est54	Estonia	DQ393773
Est3535	Estonia	DQ393774
Est2546	Estonia	DQ393779
Est3476	Estonia	DQ393776
Latvia 1–96	Latvia	AJ415565
RK1424	Latvia	AF091016
Neudoerfl	Austria	U27495
Hypr	Czech Republic	U39292
263	Czech Republic	U27491
Zausaev	Siberia, Russia	AF527415
Vasilchenko	Novosibirsk, Russia	L40361
Aina	Irkutsk, Russia	AF091006
EK-328	Estonia	DQ486861
TBEV228	Novosibirsk region, Russia	DQ385498
TBEV1467	Novosibirsk region, Russia	AY753582
Sofjin-HO	Primorskii Kray, Russia	AB062064
Oshima 5–10	Hokkaido, Japan	AB062063
LIV	United Kingdom	NC 001809
OHFV, strain Bogoluvovska	Russia	AY193805
Langat	Malaysia	AF253419
Powassan, LB strain	United States	NC 003687

*TBE, tickborne encephalitis; LIV, Louping ill virus; OHFV, Omsk hemorrhagic fever virus. †Accession no. for the partial NS5 gene sequence. ‡Accession no. for the partial E gene sequence.

## Conclusions

A TBE focus has existed in the Kokkola Archipelago at least since the 1960s when TBEV antibodies were detected in cattle (*10*), but the local TBE viruses have not been characterized. A cluster of severe human cases in the beginning of the 2000s prompted us to carry out the present study. In June 2004, we collected 1,181 ticks from the Archipelago of Kokkola and detected TBEV-RNA in 13 pools. Eleven were also positive in virus isolation. The sequences showed that the strains belonged to the Siberian subtype, whereas in the other Finnish TBE-endemic foci, only European subtype TBEV closely related to the central European strains has previously been detected (*15*). Furthermore, the tick species was *I. persulcatus*. Our results show that both *I. persulcatus* and the Siberian type TBEV are occurring several hundreds of kilometers further to northwest than what has been known previously. Because the 2 tick species are similar to the naked eye and in their behavior, and published data on distribution of *Ixodes* ticks in Finland are sparse and outdated, we cannot exclude the possibility that *I. persulcatus* also exists unnoticed elsewhere in Finland. More tick surveys and epidemiologic studies are needed to map the distribution areas of the 2 vector species and of the different TBEV subtypes in Finland. However, in our recent tick collections from Åland and southern (Isosaari, 60°N, 25°E) and eastern (Lappeenranta, 61°N, 28°E, and Joensuu, 62°N, 29°E) Finland, all ticks were *I. ricinus*.

Some researchers have found indications that the Siberian TBEV might cause more severe or more persistent forms of TBE than the European subtype (*4*), and 3 of 5 recent human TBE cases in Kokkola have been severe (*11**,**12*). However, the number of cases studied from Kokkola is too small for firm conclusions on the severity of the local disease.

## References

[1] Ecker M, Allison SL, Meixner T, Heinz FX. Sequence analysis and genetic classification of tick-borne encephalitis viruses from Europe and Asia. J Gen Virol. 1999;80:179–85. 9934700 10.1099/0022-1317-80-1-179

[2] Hayasaka D, Ivanov L, Leonova GN, Goto A, Yoshii K, Mizutani T, Distribution and characterization of tick-borne encephalitis viruses from Siberia and far-eastern Asia. J Gen Virol. 2001;82:1319–28. 11369875 10.1099/0022-1317-82-6-1319

[3] Lundkvist K, Vene S, Golovljova I, Mavtchoutko V, Forsgren M, Kalnina V, Characterization of tick-borne encephalitis virus from Latvia: evidence for co-circulation of three distinct subtypes. J Med Virol. 2001;65:730–5. 10.1002/jmv.2097 11745938

[4] Charrel RN, Attoui H, Butenko AM, Clegg JC, Deubel V, Frolova TV, Tick-borne virus diseases of human interest in Europe. Clin Microbiol Infect. 2004;10:1040–55. 10.1111/j.1469-0691.2004.01022.x 15606630

[5] Golovljova I, Vene S, Sjolander KB, Vasilenko V, Plyusnin A, Lundkvist Å. Characterization of tick-borne encephalitis virus from Estonia. J Med Virol. 2004;74:580–8. 10.1002/jmv.20224 15484275

[6] Jaenson TG, Talleklint L, Lundqvist L, Olsen B, Chirico J, Mejlon H. Geographical distribution, host associations, and vector roles of ticks (Acari: Ixodidae, Argasidae) in Sweden. J Med Entomol. 1994;31:240–56. 8189415 10.1093/jmedent/31.2.240 PMC7107449

[7] Öhman C. The geographical and topographical distribution of *Ixodes ricinus* in Finland. Acta Societatis Pro Fauna et Flora Fennica. 1961;76:1–25.

[8] International Scientific Working Group on Tick-Borne Encephalitis. 2006 Jan 11 [cited 2006 Mar 14]. Available from http://www.tbe-info.com/tbe.aspx

[9] Wahlberg P, Saikku P, Brummer-Korvenkontio M. Tick-borne viral encephalitis in Finland. The clinical features of Kumlinge disease during 1959–1987. J Intern Med. 1989;225:173–7. 10.1111/j.1365-2796.1989.tb00059.x 2703799

[10] Tuomi J, Brummer-Korvenkontio M. Antibodies against viruses of the tick-borne encephalitis group in cattle sera in Finland. Ann Med Exp Biol Fenn. 1965;43:149–54. 5893666

[11] Marjelund S, Tikkakoski T, Tuisku S, Räisänen S. Magnetic resonance imaging findings and outcome in severe tick-borne encephalitis. Report of four cases and review of the literature. Radiol. 2004;45:88–94. 15164786 10.1080/02841850410003356

[12] Marjelund S, Jääskeläinen A, Tikkakoski T, Tuisku S, Vapalahti O. Gadolinium enhancement of the cauda equina—a new MR imaging finding in a myeloradiculitic form of tick-borne encephalitis. AJNR Am J Neuroradiol. 2006;27:995–7. 16687530 PMC7975728

[13] Puchhammer-Stöckl E, Kunz C, Mandl CW, Heinz FX. Identification of tick-borne encephalitis virus ribonucleic acid in tick suspensions and in clinical specimens by a reverse transcription-nested polymerase chain reaction assay. Clin Diagn Virol. 1995;4:321–6. 10.1016/0928-0197(95)00022-4 15566853

[14] Caporale DA, Rich SM, Spielman A, Telford SR III, Kocher TD. Discriminating between *Ixodes* ticks by means of mitochondrial DNA sequences. Mol Phylogenet Evol. 1995;4:361–5. 10.1006/mpev.1995.1033 8747292

[15] Han X, Aho M, Vene S, Peltomaa M, Vaheri A, Vapalahti O. Prevalence of tick-borne encephalitis virus in *Ixodes ricinus* ticks in Finland. J Med Virol. 2001;64:21–8. 10.1002/jmv.1012 11285564

